# Effectiveness of reducing corneal astigmatism after combined high-frequency LDV Z8 femtosecond laser-assisted phacoemulsification and arcuate keratotomy

**DOI:** 10.3389/fcell.2022.1036469

**Published:** 2022-11-21

**Authors:** Hung-Yuan Lin, Shuan Chen, Ya-Jung Chuang, Suhua Zhang, Steven Wei-Hsin Chang, Pi-Jung Lin, Zhe Zhang

**Affiliations:** ^1^ Universal Eye Center, Zhong-Li, Taiwan; ^2^ Department of Ophthalmology, Shanghai Ruidong Hospital, Shanghai, China; ^3^ Department of Ophthalmology, Fujian Medical University, Fuzhou, China; ^4^ Universal Eye Center, Long-Tan, Taiwan; ^5^ Taiyuan Central Hospital of Shanxi Medical University, Taiyuan, China; ^6^ Department of Biomedical Engineering, I-Shou University, Kaohsiung, Taiwan; ^7^ Universal Eye Center, Taipei, Taiwan; ^8^ Shenzhen Eye Hospital, Shenzhen Eye Institute, Jinan University, Shenzhen, China; ^9^ The First Hospital of Shanxi Medical University, Taiyuan, China; ^10^ Shanxi Eye Hospital, Taiyuan, China

**Keywords:** high-frequency femtosecond laser-assisted phacoemulsification, arcuate keratotomy, corneal astigmatism, nomogram, cataract surgery

## Abstract

**Purpose:** In this retrospective study, the efficacy of the FEMTO LDV Z8 Femtosecond Laser-Assisted Cataract Surgery (Femto Z8 FLACS) and the Femtosecond laser Arcuate Keratotomy (FSAK) in decreasing the corneal astigmatism was investigated.

**Methods:** During FLACS, FSAK was positioned with the help of the FEMTO LDV Z8 laser at a diameter of 8.5 mm and an 80% depth. Before and 3 months after surgery, the astigmatism of the cornea was measured with the aid of Pentacam. The variables influencing the efficacy of FSAK were assessed using the multiple regression analysis technique. Vector analyses were carried out. To determine the net corneal alterations, the with-the-wound and against-the-wound variations were computed along the FSAKs’ meridian.

**Results:** This study investigated 80 eyes from 62 participants. The average keratometric astigmatism value was 0.92 ± 0.44 diopters (D). The average keratometric astigmatism decreased to 0.61 ± 0.45 D 3 months following FSAK compared to preoperative corneal astigmatism (*p* < 0.05). Additionally, there was a considerable decline in the percentage of eyes with ±0.5 D and ±1.0 D astigmatism, which reduced 3 months after surgery by 58% and 85%, respectively (*p* < 0.05).

**Conclusion:** The FEMTO LDV Z8 laser can create an effective and precise arcuate keratotomy with an excellent safety profile, rapid recovery, and vision stability.

## Introduction

The advancements in Femtosecond Laser-Assisted Cataract Surgery (FLACS) allowed ophthalmologists to achieve very accurate, safe, and predictable refractive results. At the same time, patients’ high expectations regarding “spectacle independence” have made astigmatism correction an increasingly important component of cataract surgery (Pager et al., 2004; Hawker et al., 2005). Recent large-scale retrospective surveys showed that approximately 40%–47% of eyes preparing for cataract surgery exhibit the minimal astigmatism value of 1.0 Diopter (D) (Khan and Muhtaseb, 2011; Yuan et al., 2014). Furthermore, Ferrer-Blasco et al. have reported that approximately 22% of cataract patients have 1.50 D or higher (Ferrer-Blasco et al., 2009). Villegas et al. (2014) observed a decrease in visual quality when patients showed refractive astigmatism of 0.5 D (Villegas et al., 2014). Hence, less than 0.5 D would be recommended for the visual benefit of precise astigmatism correction.

Although arcuate relaxing keratotomy is a well-established technique to manage astigmatism, most surgeons are uncomfortable with performing manual arcuate keratotomy during cataract surgery (Duffey and Leaming, 2005; Yeu et al., 2013). The femtosecond laser features precise incisions taking the depth, shape, and angulation of arcuate incisions into account, giving surgeons the ability to titrate the incision instead of creating these manually.

Notably, the femtosecond laser has received widespread recognition for its high dependability, consistency, and effectiveness in eliminating corneal astigmatism in patients suffering from mild to moderate astigmatism (Alió et al., 2014; Grewal et al., 2016; Vickers and Gupta, 2016; Baharozian et al., 2017; Roberts et al., 2018). In this review paper, Chang concluded that intrastromal FSAK is a great alternative for native eyes undergoing FLACS to correct the issue like low astigmatism (<1.5 D) and that most cuts are carried out at the optical zone of ≥7.5 mm to prevent dysphotopsia (Chang, 2018).

In planning astigmatism management, it is important to obtain reliable and consistent astigmatism measurements. Nomograms can be used to calculate arc length and optical zone required to obtain the astigmatism correction once the degree of astigmatism has been established.

Nomograms for FLACS FSAK can readily be found in recent literature (Medical; Wang et al., 2016). However. Most of the nomogram was established for high-pulses (µJ) coupled with low-frequency (kHz) laser systems, such as LenSx (Alcon Laboratories). A modified Donnenfeld nomogram has been presented by Wang et al. (2016) to support the application of the high-energy femtosecond laser platform, i.e., LenSx to correct astigmatism during cataract surgery.

The Femto LDV Z8 (Ziemer Ophthalmic Systems AG, Port, Switzerland) is a novel low-energy pulses (nJ) paired with a high-frequency (MHz) femtosecond laser system having a small spot size that pinpoints the exact location of the ocular surfaces intraoperatively. Because of this, the cutting processes in both systems are different. High-pulse energy facilitates wider spot spacing as the mechanical force applied to the expanding bubbles propels the cutting action. The low-pulse energy laser, on the other hand, allows for an increasing number of overlapping smaller-sized spots that directly vaporize the tissue inside the plasma volume, thereby successfully separating tissue without the need for the secondary mechanical tearing effects (Pepose 2008; Latz et al., 2021). As a result, the cuts achieved by low-energy pulses with high-frequency lasers, such as Femto LDV Z8 (Ziemer) system, create a smooth surface without damaging the adjacent tissues (Riau et al., 2014; Lin et al., 2021).

However, the efficacy of FSAK combined with FLACS of low-energy high-frequency laser platforms and a nomogram has not been established yet. In this study, Wang’s modified Donnenfeld nomogram was used for the purpose of estimating the number and arc length of FSAK. This study assessed the effectiveness of FSAK performed during Femto LDV Z8 FLACS. Meanwhile, a nomogram based on age and the type of astigmatism (WTR and ATR) was developed. This could be the first study that assessed the effectiveness of FSAK performed during Femto LDV Z8 FLACS (Z8 FLACS).

## Patients and methods

### Patients

In this single-center retrospective study, 80 eyes in total from 62 cataract patients who underwent the FLACS treatment between January 2019 and August 2021 were included. A skilled surgeon (HYL) at the universal Eye Center in Zhong-Li, Taiwan carried out the surgeries. The Institutional Review Board at Antai Tian-Sheng Memorial Hospital in Taiwan (21-088-B) approved the study after waiving the permission since the data was collected during routine patient care. This study was carried out following the principles of the Declaration of Helsinki for human research.

The participants who were included in this study had to fulfill the following inclusion criteria: aged ≥45 years, able to cooperate with the requirements of the docking system for femtosecond laser, should not have undergone any ocular trauma or surgery, absence of any ocular surface disease, presence of clear corneal media, ability to achieve full pupil dilation (>7 mm), and they should be able to come back for their scheduled follow-up tests. The following exclusion criteria were used in the study: minimal K-value <37 D; maximum K-value of >58 D; corneal disease or pathology with the exception of senile cataract, and a total corneal irregular astigmatism index >0.4 μm as determined by Pentacam.

### Corneal astigmatism measurements

Keratometry measurements were estimated preoperatively from Pentacam (Oculus, Wetzlar, Germany). The simulated keratometry of steep K-value, flat K-value, steep K meridian, and Km of Pentacam were measured 1 and 3 months postoperatively.

### Preoperative femtosecond laser arcuate keratotomy planning

For phacoemulsification-induced astigmatism, 0.1 D was set in the system based on an earlier analysis conducted by the same surgeon (Lin, HY M.D., data to be published). The location of the incision was the temporal side of the eyes (left eye/0° or right eye/180°). If the FSAK were closer to the primary cut, its location would be shifted 30° away from the temporal side. The arc length and number of FSAK were calculated using the Wang nomogram and were entered into the program for femtosecond laser treatment (Wang et al., 2016).

### Surgical technique

1 VERION-guided marking and manual meridian adjustment:

To prevent the effects of cyclotorsion, limbus registration was carried out with the help of a Verion image-guided system with the patients asked to sit upright. Then, a Verion system-guided 27-gage needle and ink were used and 2 endpoints of the 0°–180° horizontal axis were inscribed on the corneal limbus. When the Femto LDV Z8 image was captured after docking experiments, two blue marks at the limbus that were 180° apart could be noted clearly. The operator screen was used to project the horizontal Femto LDV Z8 reference line onto the cornea. Cyclotorsion was the cause of the angular difference between the two. The surgeon carefully set the two lines in alignment (Lin et al., 2019).

2 FLACS + FSAK technique:

Phenylephrine and tropicamide eye drops (Mydrin-P Eye Drop, Santen) were injected into the eye for 30, 25, 20, 15, and 10 min Before the procedure to help dilate the pupils. For 3 days before surgery, the patients received 4 daily injections of ketorolac 0.5% ophthalmic solution (Acular LS; Allergan, Inc., Irvine, CA). The mobile arm of the laser system was anchored over the corneal apex after the suction ring was filled with a balanced salt solution so as to form a fluid-filled interface. The femtosecond laser was used to generate a 5.5 mm capsulotomy. The lens was divided into either 4 quadrants (cataract grade II) or into 6 quadrants (cataract grade III). Following the recommendations by the manufacturer of the femtosecond laser, paired or single penetrating FSAK with a preset length were placed at an 8.5 mm diameter and a depth of 80% of the corneal thickness. The femtosecond laser produced a 1.0 mm single-plane paracentesis and a 2.2 mm primary 2-plane clean corneal incision (+40°/50°). The intraocular lenses were successfully inserted into the capsular bag.

**FIGURE 1:**
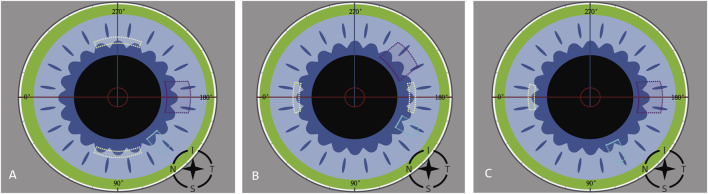
The animations from the FEMTO LDV Z8 program demonstrate the position of clear corneal incision (purple dots), paired arcuate keratotomy (yellow dots), and paracentesis (light blue dots) of FLACS with FSAK on the right eye.

In the paired FSAK group, the eyes were divided into two subgroups: With The Rule astigmatism (WTR) and Against The Rule astigmatism (ATR). For the eyes affected by WTR astigmatism, the paired FSAK was carried out around 90° and 270° based on the steep axis of corneal astigmatism (±30°). However, for eyes affected by ATR astigmatism, the paired FSAK were implemented around 0° and 180° according to the corneal astigmatism steep axis (±30°). Meanwhile, the corneal incision was shifted to 210° (OD) or 30° (OS). In a single FSAK group, for eyes affected by WTR astigmatism, one FSAK was done around 0° meridian (OD) or 180° (OS), and a clear corneal incision of FLACS was done at 180° (OD) or 0° (OS). Figure 1 depicts the animation derived from the FEMTO LDV Z8 program demonstrating the position of clear corneal incision (purple dots), paired arcuate keratotomy (yellow dots), and paracentesis (light blue dots) of FLACS with FSAK on the right eye.

### Data and statistical analysis

#### Changes in keratometry

The percentage of eyes with keratometric astigmatism that was determined with the aid of Pentacam at 0 ± 0.25 D, ± 0.50 D, ± 0.75 D, ± 1.00 D, ± 1.50 D, ± 2.00 D, and ±3.00 D before and after surgery has also been listed.

#### Astigmatism vector analysis

The Alpins method, which was included in the ASSORT software, was used to measure three vectors and their relationships while taking into consideration the differences (variations) in the keratometric astigmatism axis to study the changes occurring during the surgery (Alpins, 2001). The anticipated astigmatic modification following surgery was a target-induced astigmatism vector (TIA). Furthermore, surgically induced astigmatism (SIA) was seen to be an astigmatic change brought about by surgery. Postoperative corneal astigmatism is similar to the difference vector (DV). The ratio of SIA to TIA (SIA/TIA) is used to determine the correction index (CI). The arithmetic difference noted between the angles of the SIA and TIA is known as the angle of error (AE). The magnitude of error (ME) is the arithmetic difference between the SIA and TIA.

The flattening index (FI) shows the percentage of SIA that effectively reduces astigmatism at the target meridian (FI = SIA Cos2.AE/TIA).

#### FSAK nomograms

The Holladay-Cravy-Koch formula was used for estimating the with-the-wound (WTW) and against-the-wound (ATW) variations (Holladay et al., 1992). The Pentacam HR was used to obtain the preoperative and postoperative simulated K in addition to their axes for these computations. A WTW demonstrates the astigmatic effects along the steep corneal meridian, while an ATW reveals the astigmatic effect at 90° from the steep meridian. The WTW-ATW difference illustrates the total net effect or net corneal change induced by the incision alongside the meridian of the relaxing incision.

### Statistical analysis

SPSS software was used for all descriptive statistical analyses (version 23.0, SPSS, Inc.). The percentage data were presented in %, whereas numerical data were expressed as mean ± SD. The Kolmogorov-Smirnov test was carried out to determine whether the data distribution was normal. To assess corneal astigmatism before surgery and 3 months after surgery, a paired-sample *t*-test was employed. Statistical significance was described as a *p*-value<0.05. The net corneal changes estimated by deducting the ATW changes from WTW changes that indicate the effectiveness of FSAK were evaluated using the multiple regression analysis technique.

## Results

In total, 62 patients and 80 eyes were involved in this study. With arc lengths that ranged between 25 and 55°, 49 of the 80 eyes underwent a paired FSAK, while 31 underwent a single FSAK with arc lengths that ranged between 20 and 55°. During surgery, the average patient age was 65 ± 7 years. The average axial length was seen to be 24.4 ± 1.5 mm. The patients’ characteristics and ocular biometric parameters are listed in Table 1. No significant intraoperative or postoperative complications occurred during the surgery.

**TABLE 1:** Preoperative patient characteristics and biometric parameters.

	Mean ± SD	Range
Age (y)	65 ± 7	47 to 85
Axial length (mm)	24.4 ± 1.5	29 to 22
Pachymetry (mm)	555.9 ± 29.8	646 to 490
Pentacam Sim K		
Km vaule (D)	43.6 ± 1.4	39.5 to 47.9
Astigmatism magnitude (D)	0.9 ± 0.4	0.2 to 2.3

K, corneal power; Km, simulated keratometry; D, diopter.

### Changes in keratometric astigmatism

The percentage of eyes within ±0.5D and ±1.0 D of keratometric astigmatism measured by Pentacam significantly increased 3 months after surgery to 58% and 85%, as shown in Table 2.

**TABLE 2:** Percentage of eye within certain levels of corneal astigmatism measured by Pentacam (*n* = 80).

Keratometric Astigmatism (D)	Pre-OP keratometric Astigmatism		Post-OP 3 months keratometric Astigmatism	
Labels	Num Eyes	%	Num Eyes	%
≤0.25	2	3%	15	19%
0.26 to 0.50	8	10%	31	39%
0.51 to 0.75	23	29%	14	18%
0.76 to 1.00	22	28%	7	9%
1.01 to 1.25	7	9%	5	6%
1.26 to 1.50	11	14%	4	5%
1.51 to 2.00	5	6%	4	5%
2.01 to 3.00	2	3%	0	0%

### Astigmatism vector analysis

This study showed a significant decrease in keratometric astigmatism post-surgery. Preoperative average keratometric astigmatism or TIA value was 0.92 ± 0.44 D, which was significantly decreased to 0.61 ± 0.45 D or presented as DV 3 months postoperatively (*p* < 0.001). In paired FSAK group, the mean keratometric astigmatism was decreased from 0.93 ± 0.35 D to 0.47 ± 0.30 D at 3 months postoperatively (*p* < 0.001). However, in the single FSAK group, the mean keratometric astigmatism reduced from 0.89 ± 0.57 D to 0.83 ± 0.56 D (*p* = 0.53), with no statistical difference.

Table 3 displays the outcomes of vector analysis using the Alpins approach, including SIA, TIA, ME, DV, AE, CI, and absolute AE. In the paired FASK group, the arithmetic mean SIA magnitude was recorded to be 0.89 ± 0.54 D, which was lower than the arithmetic mean TIA in the single FSAK group. CI, which refers to the ratio of the SIA to TIA, presents the overcorrection when it is greater than one or an under correction if it is less than one. The geometric mean of CI values for paired FSAK groups was 0.84, which can be interpreted as mild under-correction, whereas the single FSAK group was 1.14, which means overcorrection.

**TABLE 3:** Vector analysis of keratometric astigmatism after femto Z8 FLACS and FSAK using the alpins method.

Vector Analysis Parameters	Pair FSAKs				
	Total (n = 80)	Total (n = 49)	WTR group (n = 34)	ATR group (n = 15)	Single FSAK (n = 31)
TIA Arithmetic mean ± SD, D Range, D	0.92 ± 0.44 0.2 to 2.3	0.93 ± 0.35 0.4 to 1.8	0.91 ± 0.34 0.5 to 1.8	0.99 ± 0.39 0.4 to 1.6	0.89 ± 0.57 0.2 to 2.3
SIA Arithmetic mean ± SD, D Range, D	0.95 ± 0.60 0.1 to 2.69	0.89 ± 0.54 0.1 to 2.57	0.72 ± 0.41 0.1 to 2.07	1.26 ± 0.61 0.33 to 2.57	1.05 ± 0.69 0.23 to 2.69
DV Arithmetic mean ± SD, D Range, D	0.61 ± 0.45 0 to 2.0	0.47 ± 0.30 0 to 1.3	0.46 ± 0.24 0.1 to 1.2	0.49 ± 0.40 0 to 1.3	0.83 ± 0.56 0.1 to 2.0
CI Arithmetic mean ± SD Geometric mean Range	1.17 ± 0.80 0.94 0.11 to 3.97	0.96 ± 0.51 0.84 0.17 to 3.17	0.80 ± 0.37 0.71 0.17 to 1.64	1.31 ± 0.63 1.21 0.67 to 3.17	−0.16 ± 0.89–1.99 to 1.77
ME Arithmetic mean ± SD, D Range, D	−0.03 ± 0.65–2.35 to 1.61	0.05 ± 0.43–1.27 to 1.15	0.19 ± 0.34–0.57 to 1.15	−0.27 ± 0.45–1.27 to 0.2	1.50 ± 1.04 1.14 0.11 to 3.97
AE Arithmetic mean ± SD, ° Range, °	1.00 ± 16.38–41.50 to 58.87	−1.78 ± 13.97–41.50 to 27.07	−2.69 ± 15.80–41.50 to 27.07	0.29 ± 8.60–15.96 to 14.57	5.39 ± 19.04–19.56 to 58.87
Absolute AE Arithmetic mean ± SD, ° Range, °	11.43 ± 11.72 0 to 58.87	9.54 ± 10.27 0 ± 41.5	10.88 ± 11.63 0 ± 41.5	6.49 ± 5.37 0 to 15.96	14.41 ± 13.33 0 to 58.87
FI Arithmetic mean ± SD, D Range, D	−1.03 ± 0.84–3.89 to 0.61	−0.87 ± 0.54–3.13 to −0.03	−0.70 ± 0.39–1.62 to −0.03	−1.26 ± 0.62–3.13 to -0.67	−1.28 ± 1.14–3.89 to 0.61
WTW-ATW Arithmetic mean ± SD, ° Range	−0.86 ± 0.63–2.67 to 0.18	−0.82 ± 0.55–2.55 to −0.02	−0.64 ± 0.43–2.01 to −0.02	−1.21 ± 0.60–2.55 to −0.32	−0.93 ± 0.74–2.67 to 0.18

TIA, target induced astigmatism; SIA, surgically induced astigmatism; DV, difference vector; ME, magnitude of error; CI, correction index; AE, angle of error; Absolute AE, absolute angle of error; FI, flattening index; WTW-ATW, net corneal change induced by the incision; SD, standard deviation.

**FIGURE 2:**
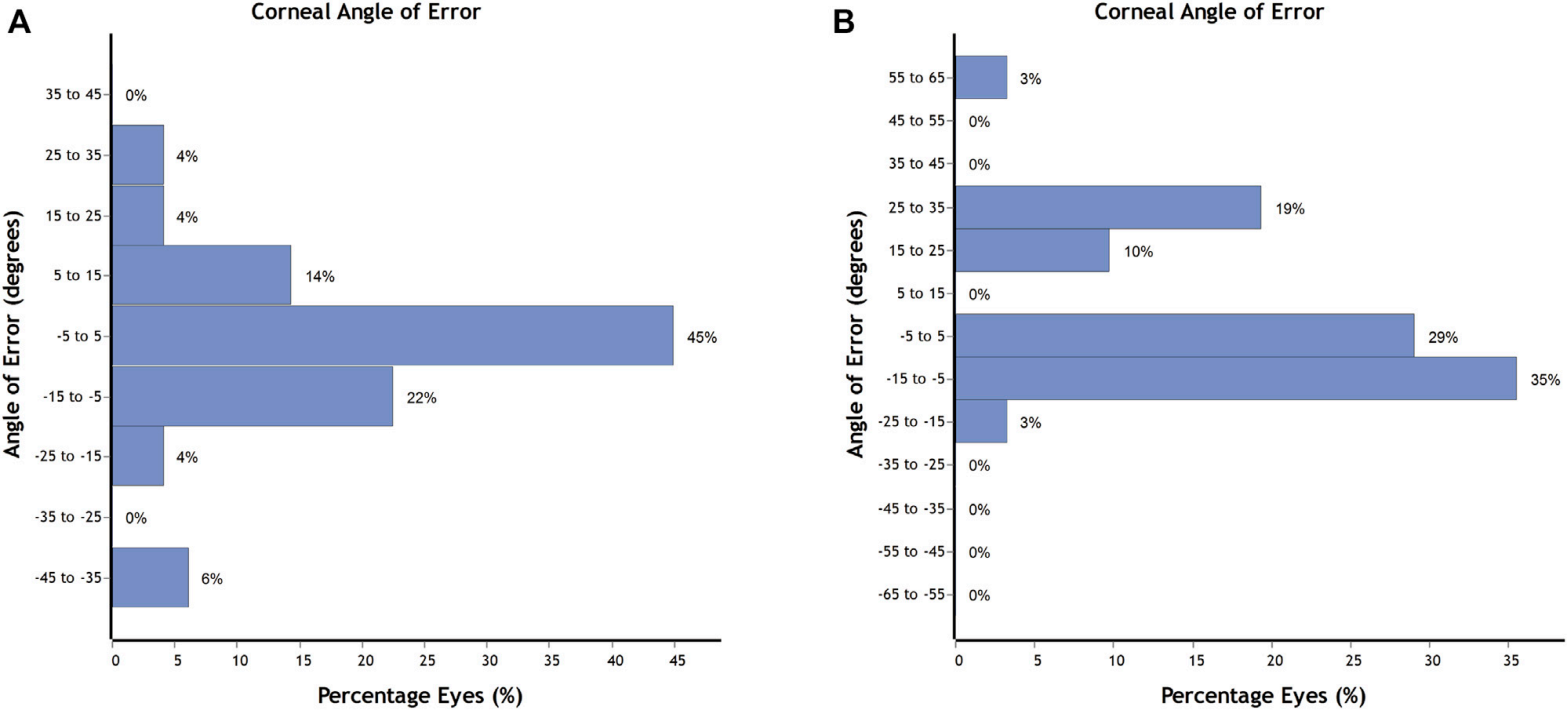
The corneal angle of error for the paired FSAK group **(A)** and single FSAK group **(B)**.

The ME (arithmetic difference present between the SIA and TIA) was −0.03 ± 0.65 D, which indicated a near-zero value or a slight under-correction. AE refers to the arithmetic variation between the angles of SIA and TIA. The corneal AE for both groups is shown in Figure 2. A total of 45% of eyes received paired FSAK during FLACS surgery and had AE within −5 to 5°, indicating no significant systematic error for the misaligned treatment. However, only 29% of eyes in the single FSAK groups had AE within ±5°, which revealed different factors, like healing or alignment at the individual level.

The FI shows the amount of astigmatism that was decreased at the intended meridian. FI shows an ideal value of −1. Here, the calculations showed an effective FI of −1.03 ± 0.84 D, which shows that astigmatism correction at that targeted orientation is effective. Significant mean net corneal changes (WTW-ATW) were −0.82 ± 0.55 D in the paired FSAK group and–0.93 ± 0.74 D in the single FSAK group, 3 months post-surgery. Whereas the magnitude of WTW-ATW astigmatism of WTR in paired FSAK group was −0.64 ± 0.43 D, ATR in the paired FSAK group was −1.21 ± 0.60 D.

**FIGURE 3:**
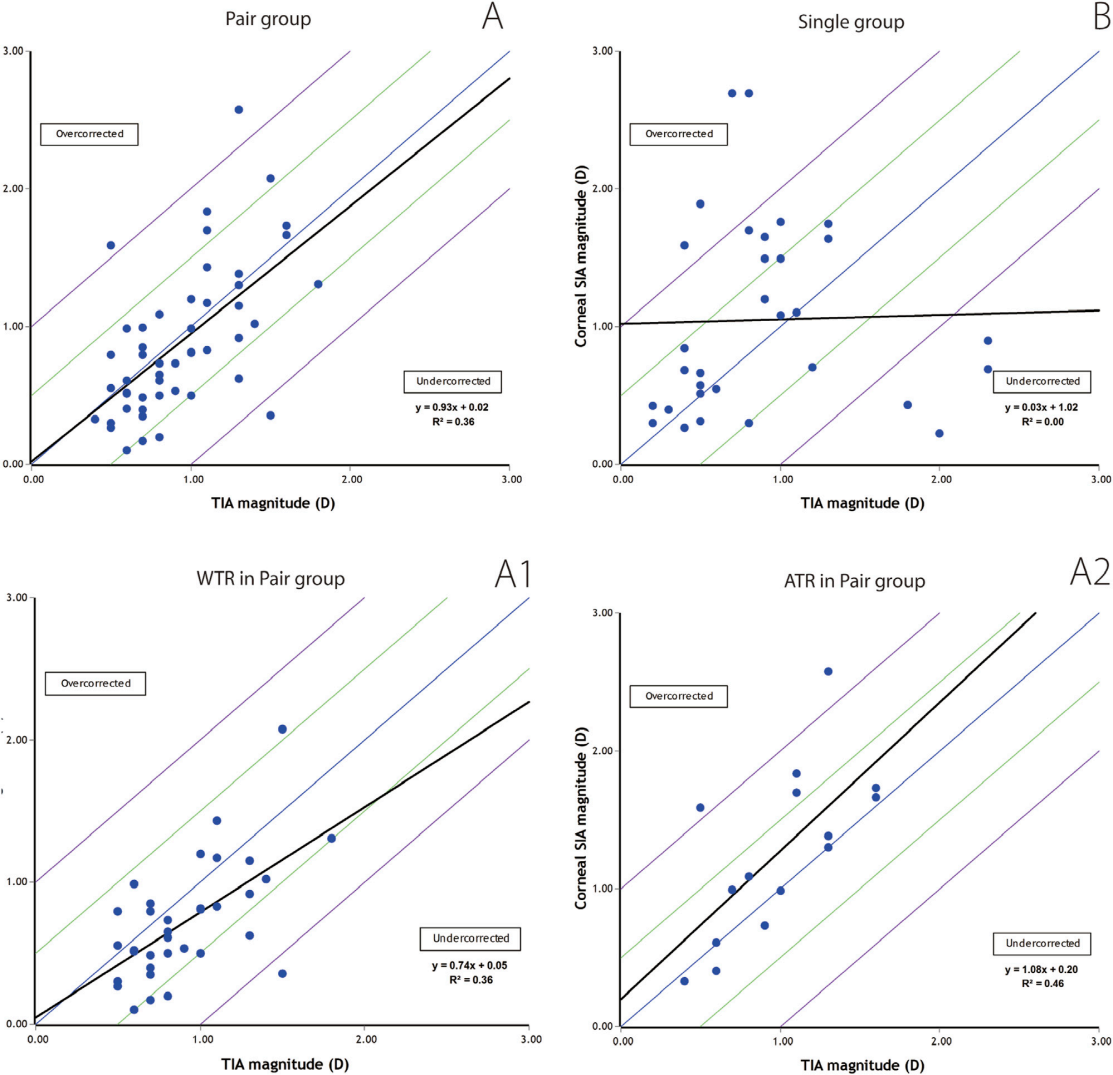
Scatterplots depicting target-induced astigmatism (TIA) vs. the surgically induced astigmatism (SIA). **(A)**. Cases of paired FSAK group; A1. Cases of WTR in paired FSAK group; A2. Cases of ATR in paired FSAK group and **(B)**. cases of single FSAK group.

A scatterplot of SIA vs. TIA after combined Femto LDV Z8 Femto and FSAK is illustrated in Figure 3. As mentioned above, overcorrection occurred when SIA/TIA is > 1, and under-correction occurred when SIA/TIA is < 1.

In paired FSAK group, the preoperative TIA and SIA values, 3 months after surgery, showed a significant relationship (r = 0.93, *p* < 0.01). However, the correlation between TIA and SIA in the single group was very low (r = 0.03, *p* = 0.886).

**FIGURE 4:**
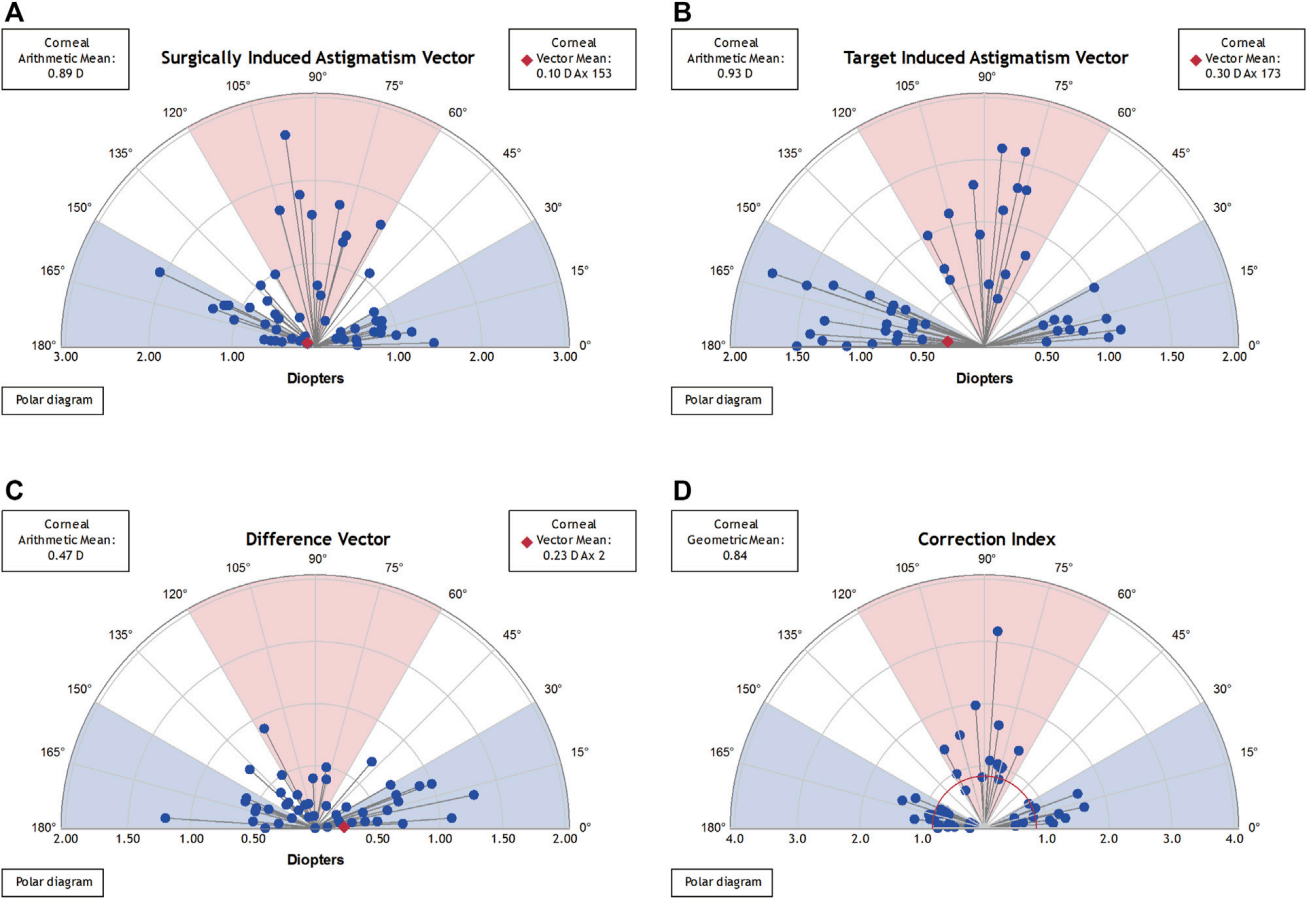
Single-angle polar plots for paired FSAK group. The vector average values were plotted in the form of a red diamond (estimated using the double-angle vector space) and the SD values for the *X* and *Y* axes were presented in a call-out box. **(A)**. SIA vector; **(B)**. TIA vector; **(C)**. DV; and **(D)**. CI.

**FIGURE 5:**
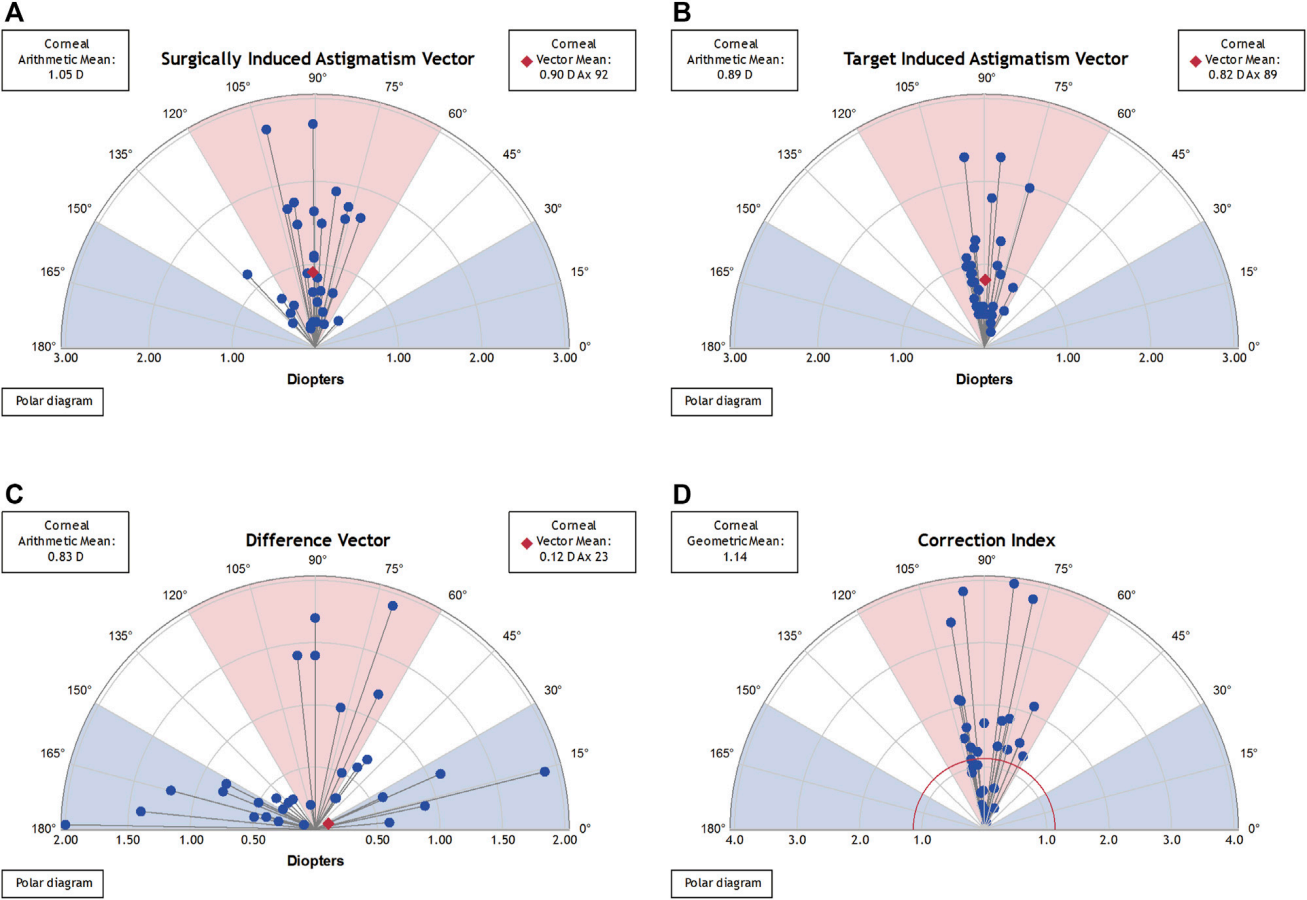
Single-angle polar plots for a single FSAK group. The vector average values were plotted in the form of a red diamond (estimated using the double-angle vector space) and the SD values for the *X* and *Y* axes were presented in a call-out box. **(A)**. SIA vector; **(B)**. TIA vector; **(C)**. DV; and **(D)**. CI.

Figures 4, 5 present the single-angle polar plots for vector TIA, DV, vector SIA, and CI for both groups. The standard deviation for the X and Y axes was visualized in the call-out box, and vector means are represented as red diamonds (measured in the double-angle vector space).

### Femtosecond laser arcuate keratotomy nomograms

The net corneal changes (WTW-ATW) were derived for nomogram development using the corneal simulated variations that were estimated using the Pentacam 3 months following surgery (Table 4). According to the age and length of the paired FSAK, the following regression formulas were used for eyes having WTR and ATR corneal astigmatism:

**TABLE 4:** Nomogram: total net corneal change (WTW-ATW changes) based on age and length of FSAKs in paired group with WTR and ATR corneal astigmatism.

Paired Incision Length	Age							
		50 years	55 years	60 years	65 years	70 years	75 years	80 years
WTR eyes								
	25°	0.15	0.07	−0.01	−0.09	−0.17	−0.25	−0.33
	30°	−0.06	−0.14	−0.22	−0.30	−0.38	−0.46	−0.54
	35°	−0.27	−0.35	−0.43	−0.51	−0.59	−0.67	−0.75
	40°	−0.47	−0.55	−0.63	−0.71	−0.79	−0.87	−0.95
	45°	−0.68	−0.76	−0.84	−0.92	−1.00	−1.08	−1.16
	50°	−0.89	−0.97	−1.05	−1.13	−1.21	−1.29	−1.37
	55°	−1.09	−1.17	−1.25	−1.33	−1.41	−1.49	−1.57
	60°	−1.30	−1.38	−1.46	−1.54	−1.62	−1.70	−1.78
ATR eyes								
	25°	−0.52	−0.60	−0.68	−0.76	−0.84	−0.92	−1.00
	30°	−0.73	−0.81	−0.89	−0.97	−1.05	−1.13	−1.21
	35°	−0.94	−1.02	−1.10	−1.18	−1.26	−1.34	−1.42
	40°	−1.14	−1.22	−1.30	−1.38	−1.46	−1.54	−1.62
	45°	−1.35	−1.43	−1.51	−1.59	−1.67	−1.75	−1.83
	50°	−1.56	−1.64	−1.72	−1.80	−1.88	−1.96	−2.04
	55°	−1.76	−1.84	−1.92	−2.00	−2.08	−2.16	−2.24
	60°	−1.97	−2.05	−2.13	−2.21	−2.29	−2.37	−2.45

Paired group:

a) WTRNet corneal changes = −0.01601507 × age (years) − 0.0413699 × length (degrees) + 1.98254955
b) ATRNet corneal changes = −0.01601507 × age (years) − 0.0413699 × length (degrees) − 0.66906866 + 1.98254955.

In the single FSAK group, the correlation between TIA and SIA was very low. Therefore, a nomogram for the single FSAK was not designed (r = 0.03, *p* = 0.886).

## Discussion

Two alternative laser parameter patterns are used by FLACS: high-energy pulses (µJ) coupled with a low-frequency (kHz); low-energy pulses (nJ) coupled with a high-frequency (MHz). Because of this, the cutting processes used by both systems are different. A particularly smooth surface is produced by the Femto LDV Z8 (Ziemer) system without damaging the surrounding tissues (Riau et al., 2014; Lin et al., 2021). This is important for the FSAK because it offers smoother corneal incisions during surgery. The other difference between the LenSx and Femto LDV Z8 systems is the docking interface. Femto LDV Z8 system employs a liquid-filled interface, where the vacuum ring improves the contact with the sclera, and the center is filled with liquid. LenSx system uses an applanating curved interface, which directly touches the cornea. Though during the surgery, the mechanical contact interface significantly stabilizes the cornea, the use of a liquid-filled interface has been found to prevent corneal folds that may cause incomplete capsulotomy.

Yet, as mentioned above, the efficacy of FSAK combined with FLACS of low-energy high-frequency laser platforms could be different. Each platform has developed its recommendations to minimize unwanted problems during the FSAK procedure. According to the manufacturer’s instructions, the paired FSAK was placed for the Femto LDV Z8 at a diameter of 8.5 mm and a corneal thickness depth of 80%. The modified Donnenfeld nomogram proposed by Wang et al. (2016) was used to calculate the number and length of FSAK since there was no calculator available to do so during FLACS.

In this study, vector analysis showed that the mean keratometric astigmatism measured by Pentacam was decreased in the 3rd month after surgery. The vector analysis was conducted to estimate the WTW and ATW changes that denote the overall net effect caused by the incisions. At 3 months after surgery, the average WTW and ATW changes in the paired FSAK group were recorded to be −0.82 ± 0.55 D.

The multiple regression analysis revealed that age, FSAK location, and FSAK arc length were found to significantly influence total net corneal modifications (WTW-ATW changes). Age, larger incisions, horizontal incisions (0° or 180°), corneal astigmatism, and preoperative ATR all contribute to the amount of net corneal modifications that arise in eyes with preoperative ATR. In this study, the main incision of FLACS was at the temporal side of the cornea, which is more stable and predictable than the oblique angle, such as 120° or 60°.

Based on the results obtained in this study, the effectiveness of FSAK alongside the horizontal meridian is greater than the vertical meridian within the same age groups. This could be attributed to corneal biomechanical factors linked to temporal incision. As a result, it was seen that the length of arcuate incision needed for ATR cornea is less than WTR cornea for the same correction of astigmatism. Previous studies have shown that the amplitude of refractive cylinder increases with increasing age, and its orientation also shifts from WTR to ATR (Saunders, 1986; Gudmundsdottir et al., 2000; Navarro et al., 2013; Rozema et al., 2019). Thus, for long-term FLACS and FSAK surgical outcomes, it is suggested that it is better to have overcorrection than under-correction when dealing with ATR corneal astigmatism.

In terms of the single FSAK group in this study, as the correlation between TIA and SIA was very low and did not show statistical significance, there is no reliable astigmatism reduction. When considering the effectiveness, the asymmetrical incisions created by FSAK and FLACS could lead to variable biomechanical responses, and therefore, variable effectiveness in reducing ATR astigmatism in a single FSAK group. The incisions of FSAK and FLACS could result in variable influences on several aspects, such as the angle of the corneal incision, full/partial depth, and one/two plans.

This study has a few drawbacks. Firstly, the sample size is small, necessitating more extensive patient follow-up visits. The purpose of this study was to present the preliminary findings and recommend a Z8 nomogram. More patients will be included in the trial with a longer follow-up duration, and the nomogram’s performance will be assessed further. Secondly, the keratometric and refractive results of a small subset of eyes that underwent single FSAK were merged with those of the eyes that experienced paired FSAK. These conclusions sum up the outcomes of these related cases. Eyes were separated into single FSAK and paired FSAK groups to compute and visualize the WTW and ATW alterations induced by the FSAK.

In conclusion, low-energy high-frequency femtosecond laser arcuate keratotomy performed during cataract surgery can reduce the pre-existing low-grade corneal astigmatism in a short-term observation. Long-term observation of refractive stability is needed. Furthermore, our nomogram needs to be refined, requiring more cases to be enrolled in the future. It is necessary to evaluate the length and depth of the incisions and determine the role played by the corneal biomechanical factors to assess the long-term benefits.

## Data Availability

The raw data supporting the conclusion of this article will be made available by the authors, without undue reservation.
